# National approaches to managing cancer care: responses of countries in the MENA region to the COVID-19 pandemic

**DOI:** 10.3332/ecancer.2021.1189

**Published:** 2021-02-23

**Authors:** Zineb Benbrahim, Layth Mula-Hussain, Humaid O Al-Shamsi, Nagi El Saghir, Mushabbab Al Asiri, Bassim Al Bahrani, Muath Al Nassar, Adda Bounedjar, Zahera Fahed, Sami Khatib, Ola Khorshid, Soumaya Labidi, Nawfel Mellas, Amani Saleh, Abdulrahman Jazieh

**Affiliations:** 1 Hassan II University Hospital, Faculty of Medicine and Pharmacy of Fez, University Sidi Mohamed Ben Abdellah, Fez, Morocco; 2 College of Medicine, Ninevah University, Mosul, Iraq; 3 University of Sharjah, Sharjah, UAE; 4 American University of Beirut, Beirut, Lebanon; 5 Saudi Cancer National Institute, Riyadh, Saudi Arabia; 6 King Fahad Medical City, Riyadh, Saudi Arabia; 7 National Oncology Center, Royal Hospital, Muscat, Oman; 8 Kuwait Cancer Control Center, Kuwait City, Kuwait; 9 Laboratoire de cancérologie, Faculté de Médecine, Université Blida 1, Blida, Algeria; 10 St Louis Hospital, Damascus, Syria; 11 Dar Al Shifaa Hospital, Damascus, Syria; 12 Private Sector, Amman, Jordan; 13 NCI Cairo University, Cairo, Egypt; 14 Medical Oncology Department SOMA, Abderrahmane Mami Hospital, Ariana, Tunisia; 15 Faculty of Medicine, Tunis El Manar University, Tunis, Tunisia; 16 National Oncology Center, Aden, Yemen; 17 King Abdullah International Medical Research Center, King Saud bin Abdulaziz University for Health Sciences, National Guard Health Affairs, Riyadh, Saudi Arabia

**Keywords:** cancer care, MENA region, COVID-19

## Abstract

**Background:**

The coronavirus disease 2019 (COVID-19) pandemic presents serious challenges to cancer care because of the associated risks from the infection itself and the disruption of care delivery. Therefore, many professional societies have published recommendations to help manage patients with cancer during the current pandemic. The objective of our study is to assess the national responses of Middle East North Africa (MENA) countries in terms of publishing relevant guidelines and analyse various components of these guidelines.

**Methods:**

A survey based on the preliminary review of the literature regarding cancer care adaptations has been developed and then completed by a group of oncologists from the following Arab countries affected by the pandemic: Algeria, Egypt, Iraq, Jordan, Kuwait, Lebanon, Morocco, Oman, Saudi Arabia, Syria, Tunisia, United Arab Emirates and Yemen. The survey inquired about COVID-19 cases, national recommendations regarding general measures of COVID-19 prevention and patient care in oncology as well as their implementation about cancer care adaptations during the pandemic.

**Results:**

Analysis of the COVID-19 pandemic-related guidelines revealed at least 30 specific recommendations that we categorised into seven essential components. All included countries had national guidelines except one country. Estimated full compliances with all specific category recommendations ranged from 30% to 69% and partial compliance ranged from 23% to 61%.

**Conclusion:**

There is a very good response and preparedness in the Arab Middle East and North Africa region surveyed. However, there are inconsistencies in the various components of the guidelines across the region, which reflects the evolving status of the pandemic in each country as well as the lack of clear evidence-based guidelines for many of the issues in question. There is a need for a clear framework on essential components that should be included in these guidelines to assure providing the best guidance to the oncology community.

## Background

Since its first report in China in December 2019, the severe acute respiratory syndrome (SARS) caused by the novel coronavirus disease 2019 (COVID-19) has resulted in more than 47,658,000 infections and 1,215,000 deaths by 3 November 2020 [1].

COVID-19 has been reported in all Middle East North Africa (MENA) countries. The MENA region accounts for 6% of the world’s population. It has no standardised definition, however, it most commonly covers the Gulf Cooperation Council (GCC) countries (Bahrain, Kuwait, Oman, Qatar, Saudi Arabia and the United Arab Emirates (UAE)), Levant region (Jordan, Lebanon, Palestine and Syria), North Africa (Algeria, Libya, Morocco and Tunisia), Egypt, Iraq and Yemen. All these countries are different at social and economic levels with GDPs ranging from 14,616 (Gaza, part of Palestine) to 782,483 million US$ (Saudi Arabia) [2].

Since the first case was detected in the UAE on 29 January 2020, a sharp increase in cases and deaths was recorded. On the one hand, all these countries are taking precaution measures in order to control the spread of COVID-19 and the overwhelming of health services by closing borders, public places and schools, cancelling congregational prayers, etc [3, 4]. On the other hand, the MENA economies’ ability to respond was different from the point of view of the GCC, compared to the Levant or North Africa, or in the war-affected countries. Indeed, many of the public health and hospital-based interventions deployed by high-income countries were infeasible in low-income countries [5]. One feature of these discrepancies was the significant shortage of personal protective equipment, the drug shortage in limited-resource-countries and even the reduction of overseas aid from high-income countries [6]. One indicator of the variance of deployed measures during the COVID-19 pandemic is the case fatality ratio which ranged, by 3 November, from 0.37% in UAE to 29.13% in Yemen.

Cancer is a public health issue in all MENA countries with different prevalence affecting from 60 to 216 per 100,000 of the populations [7]. Management of cancer in the MENA region faces a great difference mainly with regard to lack of comprehensive up-to-date population registries, lack of human resources, medical equipment and access to novel drugs in low-income countries. COVID-19 exacerbated inequity in cancer care in the MENA region. Hospital systems were even more overloaded in less prepared countries. Travel restrictions have resulted in many cancer outpatient visits and chemotherapy delays especially in countries where major health facilities and cancer centres are centralised in some cities.

Several data are suggesting susceptibility of COVID-19 infection and occurrence of severe events in patients with cancer. Actually, according to a Chinese study, cancer patients represented 1% of the COVID-19 infected patients, whereas the incidence of cancer in the general population was 0.29% [8]. Additionally, the case fatality rate among cancer patients exceeded largely the rates retrieved in the general population (5.6% versus 2.3%, respectively) [9].

Paradoxically, there is a limitation of scientific data supporting approaches to managing cancer patients in this pandemic whereas oncological decisions are used to be carefully based on concise information deduced from large clinical trials. Within this absence of guidance, international professional oncological societies [10, 11], as in the MENA region, had to step in and publish new recommendations aiming to implement preventive measures to protect cancer patients as well as healthcare staff while ensuring continuity of cancer care. Most adopted guidelines have been based on expert opinions and supported by data extrapolated from previous outbreaks.

In this study, our main objective was to assess the national responses of MENA countries in terms of publishing relevant guidelines and analyse various components of these guidelines. This assessment was intended through approaching senior oncologists from each of the 17 countries of the MENA region (Algeria, Bahrain, Egypt, Iraq, Jordan, Libya, Kuwait, Lebanon, Morocco, Oman, Palestine, Qatar, Saudi Arabia, Syria, Tunisia, UAE and Yemen).

## Methods

### Instrument development

The instrument that was used to address this study’s objectives is a survey that has been constructed in two stages:

Stage 1: The survey has been developed based on the preliminary review of the literature about health care adaptations regarding clinical activities, health care workers (HCW) management, reorganisation of cancer centres and cancer-specific treatments administration and existing guidelines [12–19].

Stage 2: We intended to approach one senior oncologist from each of the 17 MENA countries. All the 13 following countries have been represented: Algeria, Egypt, Iraq, Jordan, Kuwait, Lebanon, Morocco, Oman, Saudi Arabia, Syria, Tunisia, UAE and Yemen. However, no experts were identified from Libya and Palestine (because of lack of contact with collaborators) and Qatar (because of lack of time to obtain approval of the Institutional Review Board (IRB) to perform such study). All the 13 approached senior oncologists reviewed the questionnaire for clarity, appropriateness and relevancy of each item discussed. A cross-check of the survey was done by all the identified experts to appraise its content validity. Finally, the modified final survey has been completed based on each country’s information collected on 3 November 3.

### Instrument content

The survey questioned about instructions of the national recommendations in five parts:

- Part 1: Nine items about the pandemic situation of the COVID-19 infection in the MENA countries. Data about COVID-19 cases were collected from the Center for Systems Science and Engineering website of the Johns Hopkins University (JHU) [1]. Details related to the lockdown declaration in different countries were acquired from online media resources [20]. Partial or whole country lockdown was determined on the basis of geographical extent of the lockdown in each country (some regions or all the country).
- Part 2: Six items about the availability of national recommendations on practice-changing of cancer care during the COVID-19 pandemic.
- Part 3: Four items about instructions of the national recommendations regarding general measures of COVID-19 prevention in oncology in the MENA region.
- Part 4: Three items about instructions of the national recommendations regarding cancer care adaptations during the pandemic.
- Part 5: Two open-ended items asking about what should have been done differently if the scenario repeated and what should be the permanent changes that should be integrated into oncology care to minimise the risk of future pandemics.

All the variables in each part of the questionnaire were identified, and then we created a comprehensive checklist of all items. Availability of instructions about each item has been verified for each country’s guidelines. Data were analysed to highlight the different responses that have been adapted and the main challenges that are faced in the region. Descriptive statistics were used to examine study variables (mainly to determine frequency of compliance of National Guidelines to Recommendation).

## Results

By 3 November, the estimated number of COVID-19 infected cases in the MENA countries was at least 1,849,629 cases representing almost 4% of the total number of detected cases in the globe (47,658,000 cases detected worldwide according to the JHU on 3 November). This number varied from 2,063 in Yemen to 482,296 in Iraq with different rates of fatality. The highest death percentages were observed in Yemen, whereas the lowest was in the UAE. Table 1 illustrates epidemiological data about COVID-19 pandemic in the MENA countries.

Most cancer epidemiological data in MENA countries are issued from population-based cancer registries. Sources of epidemiologic data in the remaining countries include estimations from international registries (Globocan 2018).

Among the 13 countries included in this study, 9 (Algeria, Egypt, Iraq, Kuwait, Lebanon, Morocco, Saudi Arabia, Syria and Tunisia) issued national guidelines for practice-changing of cancer care during the COVID-19. In one country (Jordan), practice-changing was based on institutional guidelines whereas no local guidelines have been issued in three countries (Oman, UAE, Yemen) where oncologists are guided by international recommendations. Analysis of the guidelines revealed at least 30 recommendations categorised into seven essential components with specific recommendations for each component.

### Regarding general measures of COVID-19 prevention in oncology in the MENA region

#### Patient management

All the approached MENA countries that issued guidelines (*N* = 12) endorsed screening patients with cancer for COVID-19 symptoms before their admission. Providing surgical masking for all patients and postponing routine follow up visits were also recommended by the majority of these countries guidelines (*N* = 11) while nine of these guidelines suggested implementing call centres to triage patients.

*HCW management*All the approached MENA countries (*N* = 12), at the unanimity, issued guidelines about educating HCW to adhere to standard precautions, wearing a mask during consultations and disinfecting hands regularly. Ten guidelines recommended N95 fitting while staff clustering was endorsed by less than half of the countries’ guidelines.

#### Facility management

Restricting visitors, keeping distance of 1.5 m between each two patients, disinfecting work surfaces and any instrument used after the examination of each patient are measures endorsed by 11 MENA countries guidelines while the 12 countries recommended establishing a plan of action for COVID-19 suspected cases.

### Regarding cancer care adaptations during the pandemic

All the approached MENA countries (*N* = 12) at the unanimity issued guidelines about postponing non-essential para-clinical examinations. The majority (*N* = 12) recommended favouring surveillance by teleconsultation in selected situations, favouring oral treatments and avoiding weekly schedules whenever possible. Hypofractionating radiotherapy was endorsed by nine MENA countries guidelines.

Sites specific recommendations and other measures reducing COVID-19 complications (like reducing the use of corticoids whenever possible and using granulocytic growth factors if the risk of febrile neutropenia exceeds 10%) were endorsed by only six of the approached MENA countries.

Tables 2 and 3 show compliance of each MENA country’s guidelines with having these recommendations.

## Discussion

### Summary of results and comparison to findings from previous research

Despite the gap between MENA countries in facing COVID-19 pandemic, and based on initial data from Chinese and Italian reports [21–22] suggesting a higher risk of COVID-19 infection, most countries in MENA region established local national or institutional guidelines in caring for cancer patients during the pandemic period. Given the paucity of high-level evidence at this point of the COVID-19 pandemic, most proposed guidelines have been based on expert opinions.

It is crucial to note that cancer patients are immunocompromised and are at increased risk of COVID-19-related deaths [13]. This was reported first in a Chinese study concluding that cancer patients with COVID-19 who received anticancer therapy in the preceding 14 days of COVID-19 diagnosis had more than four-fold higher likelihood of experiencing severe events [23]. Extra precaution is very much recommended for this population, thus guidelines in the MENA countries suggested multiple dimensions during COVID-19 pandemic to protect patients and HCW. These guidelines, at the quasi-unanimity, insisted on the main measures recommended by the World Health Organisation (WHO) [24], the American Society of Clinical Oncology (ASCO) [6] and others [12–19]. However, there was no unanimous recommendation about N95 fitting since the WHO stresses the need for prioritising medical masks and respirators for HCW [25].

Regarding cancer-specific care adaptations during the pandemic, the majority of oncology societies in MENA countries issued guidance to convert intravenous treatments to oral or subcutaneous regimens whenever possible, to avoid weekly schedules, to space immunotherapy cycles and switch radiotherapy to hypofractionated regimens to limit hospital admission of patients. Postponing non-essential para-clinical examinations and favouring surveillance by teleconsultation where possible were also suggested by these guidelines. However, it is important to notice some differences in specific strategies such as using granulocytic growth factors whenever the risk of febrile neutropenia exceeds 10% or reducing the use of corticoids.

- Actually, the use of Granulocyte-Colony Stimulating Factor (G-CSF) has been supported by some guidelines to boost the immune system while others worried the lymphocytic response decrease in case of COVID-19 infection. Additionally, an excessive and uncontrolled release of pro-inflammatory cytokines such as G-CSF levels has been reported to be higher in infected patients with COVID-19 requiring intensive care [26, 27].
- Regarding corticosteroids use, evidence for harm from these drugs have been concluded from previous studies reported during the outbreaks of Middle East respiratory syndrome (MERS) or SARS. Indeed, a retrospective observational study reporting 309 infected adults with MERS concluded to a delay in the clearance of viral RNA from respiratory tract secretions in patients given corticosteroids [28]. Also, a meta-analysis identified a total of 6,548 patients with influenza with increased mortality in patients who were given corticosteroids [29]. In opposition, a Chinese study concluded to most favourable SARS-CoV patient outcomes in those treated early with high dose steroids in combination with a quinolone [30].

These conflicting data in the existing literature does not currently provide conclusive evidence for or against the use of GCSF or corticosteroids and reflect the different attitudes of oncological guidelines.

### Strengths and limitations of the study

This study reported for the first time a thorough look of MENA region response during the COVID-19 pandemic and might offer guidance for the oncology community during future outbreaks. Nevertheless, some limitations of this report should be noted:

One of the limitations is not having the full picture about the pandemic status in each country due to the variation in testing and discrepancy in reporting in the midst of the pandemic that is rapidly evolving. Another limitation of this study was the dependence on the consensus of expert’s opinion and not on evidence standards of care but that will be the most practical approach with new crisis until evidence emerges and incorporates into practice.

Therefore, all the listed instructions should be re-discussed according to the available data in the literature and should be adapted to the evolution of the health situation in each territory of the MENA region.

### Implications for future cancer research, practice and policy

The COVID-19 pandemic is currently a public health crisis that has posed unprecedented challenges in the management of cancer patients in the MENA region. During such crises, the generation of timely management recommendations was crucial.

According to this study, there is inconsistency in the components of the guidelines across the MENA region, which may reflect the evolving nature of the pandemic and lack of clear evidence for many of the issues in question. There is a need from a clear framework on essential components that should be included in the guidelines to assure providing the best guidance to the oncology community.**Conclusion**

Finally, it is important to highlight that professional oncology societies in the MENA region should carefully consider management modifications carried out during this pandemic as an opportunity for cancer care in the future as conducting research comparing therapeutic standards with de-escalated regimens during this pandemic may be very informative. This might be accomplished through a platform gathering oncology leaders from different countries within the MENA region. Afterwards, expertise may be shared across borders among MENA countries and with international organisations (WHO, NCCN).

## Authors’ contributions

ZB and AJ reviewed the literature and constructed the survey, designed the study, collected data and wrote the manuscript.

LM, HA, NE, MA, BA, MA, AB, ZF, SK, OK, SL, NM and AS reviewed and cross checked the survey, collected data and revised the manuscript.

All authors approved the manuscript before submission.

## Conflicts of interest

Zineb Benbrahim received educational grants from Novartis, MSD, AstraZeneca. Humaid O Al Shamsi received research support from ROCHE. AR Jazieh received research funds from MSD.

## Funding statement

No funding was received for this research

## Figures and Tables

**Table 1.** Epidemiological overview of the COVID-19 pandemic in MENA countries.

Country	Total population	Total cancer cases	Date of first confirmed COVID-19	Total number of cases by 3 November	Total number of deaths by 3 November	Fatality rate (%)	Period of lockdown	Extent of lockdown
Algeria	42,230,000	53,076	25 Feb	58,979	1,980	3.35	15 March to 28 July	Partial
Egypt	100,000,000	128,892	14 Feb	107,925	6,291	5.83	25 March to 27 June	Whole
Iraq	39,339,754	31,502	24 Feb	482,296	11,068	2.29	17 March to 18 April	Whole
Jordan	6,879,875	5,999	15 Feb	86,576	967	1.11	18 March to 30 April	Whole then partial
Kuwait	4,137,312	5,804	24 Feb	128,080	789	0 .61	10 May to 31 May	Whole
Lebanon	6,000,000	12,000	19 Feb	85,209	667	0.78	13 April to 13 May	Whole
Morocco	36,472,000	52,783	2 March	229,565	3,900	1.70	12 March to 24 June	Whole country
Oman	4,829,473	3,322	24 Feb	116,528	1,264	1.08	10 April to 29 May	Partial
Saudi Arabia	33,554,333	24,485	2 March	348,510	5,456	1.56	23 March to 21 June	Whole country
Syria	18,284,423	23,170	23 March	5,843	295	5.04	23 March to 26 May	Whole
Tunisia	11,582,075	15,894	2 March	61,906	1,381	2.2	20 March to 8 June	Whole
UAE	9,631,000	4,000	29 Jan	136,149	503	0.37	4 April to 17 June	Partial
Yemen	28,500,000	-	9 April	2,063	601	29.13	17 May to 17 July	
Total	341,440,245	>360,927	-	1,849,629	35,162	1.9	-	-

**Table 2.** National guidelines regarding general measures of COVID-19 prevention in oncology in the MENA region.

Recommended measures	AL	EG	IQ	JO	K.S.A	KW	LB	MR	OM	SY	TN	UAE	YE
**Patient management**													
Screening of COVID-19 symptoms before clinic (outpatients) visit	**+**	**+**	**+**	**+**	**+**	**+**	**+**	**+**	**+**	**-**	**+**	**-**	**-**
Screening of COVID-19 symptoms before admission of patients	**+**	**+**	**+**	**+**	**+**	**+**	**+**	**+**	**+**	**+**	**+**	**+**	**-**
Providing surgical masking for all patients	**+**	**+**	**-**	**+**	**+**	**+**	**+**	**+**	**+**	**+**	**+**	**+**	**-**
Postponing routine follow up visits	-	+	+	+	**+**	**+**	**+**	**+**	**+**	**+**	**+**	**+**	**-**
Implementing call centres to triage patients	**+**	**-**	**+**	**+**	**+**	**+**	**+**	**+**	**+**	**-**	**+**	**-**	**-**
**HCW management**													
Favouring tumour board meetings based on videoconferencing	**+**	**+**	**-**	**+**	**+**	**+**	**+**	**+**	**+**	**-**	**+**	**-**	**-**
Educating HCW to adhere to standard precautions	**+**	**+**	**+**	**+**	**+**	**+**	**+**	**+**	**+**	**+**	**+**	**+**	**-**
Recommending HCW to:													
Wear a mask during consultations	**+**	**+**	**+**	**+**	**+**	**+**	**+**	**+**	**+**	**+**	**+**	**+**	
Disinfect hands regularly	**+**	**+**	**+**	**+**	**+**	**+**	**+**	**+**	**+**	**+**	**+**	**+**	**-**
N95 fitting	**+**	**+**	**+**	**-**	**+**	**+**	**+**	**-**	**+**	**+**	**+**	**+**	**-**
Staff clustering	**-**	**-**	**+**	**-**	**+**	**+**	**-**	**-**	**+**	**-**	**+**	**-**	**-**
**Facility management**													
Restricting visitors	**+**	**+**	**-**	**+**	**+**	**+**	**+**	**+**	**+**	**+**	**+**	**+**	
Establishing screening and triage stations at entrance of cancer centres units	**+**	**+**	**+**	**+**	**+**	**+**	**+**	**+**	**+**	**-**	**+**	**-**	**-**
Keeping distance of 1.5 m between each two patients	**+**	**+**	**-**	**+**	**+**	**+**	**+**	**+**	**+**	**+**	**+**	**+**	**-**
Disinfecting any instrument used after the examination of each patient	**+**	**+**	**-**	**+**	**+**	**+**	**+**	**+**	**+**	**+**	**+**	**+**	**-**
Disinfecting work surfaces regularly	**+**	**+**	**-**	**+**	**+**	**+**	**+**	**+**	**+**	**+**	**+**	**+**	**-**
Establishing a plan of action for suspected cases	**+**	**+**	**+**	**+**	**+**	**+**	**+**	**+**	**+**	**+**	**+**	**+**	**-**
**Testing for COVID-19**													
Real time PCR and chest CT scan	**+**	**+**	**+**	**+**	*	**+**	**+**	**+**	**+**	**-**	**+**	**-**	**-**
Specifying whom to test	**+**	**+**	**+**	**+**	*	**+**	**+**	**+**	**+**	**-**	**+**	**-**	**-**
Specifying what to do with cancer patients with positive test	**+**	**-**	**+**	**+**	**+**	**+**	**+**	**+**	**+**	**+**	**+**	**+**	**-**

AL: Algeria, EG: Egypt, IQ: Iraq, JO: Jordan, KW: Kuwait, LB: Lebanon, MR: Morocco, OM: Oman, K.S.A: Saudi Arabia, SY: Syria, TN: Tunisia, UAE: United Arab Emirates, YE: Yemen

+: Item discussed in the country guidelines, -: Item non discussed in the guidelines

**Table 3.** Recommendations of the National guidelines regarding cancer care adaptations during the pandemic.

Recommended measures	AL	EG	IQ	JO	KSA	KW	LB	MR	OM	SY	TN	UAE	YE
**Measures to reduce hospital visits**													
Favouring oral treatments	+	+	+	+	+	-	+	+	+	+	+	+	+
Avoiding weekly schedules whenever possible	+	+	+	+	+	+	+	+	+	+	+	-	+
Spacing of immunotherapy cycles	+	-	+	+	+	+	+	+	+	+	NA	-	NA
Hypofractionating radiotherapy	-	-	+	+	+	+	+	+	+	+	+	-	NA
Favouring surveillance by teleconsultation in selected situations	+	+	+	+	+	+	+	+	+	-	+	+	-
Postponing non-essential para-clinical examinations	+	+	+	+	+	+	+	+	+	+	+	+	-
**Other mesures to reduce complications**													
Reducing the use of corticoids, whenever possible	+	+	+	-	-	_	+	+	-	+	_	-	-
Using granulocytic growth factors if the risk of febrile neutropenia exceeds certain percentage (10% ?)	+	+	-	-	-	+	+	+	-	+	_	-	-
**Site specific recommendations**													
Breast cancer	-	-	-	+	+	+	+	+	+	+	_	-	_
Lung cancer	-	-	-	+	+	+	+	+	-	+	_	+	-
GI cancers	-	-	-	+	+	+	+	+	+	+	_	-	-
Gynaecologic cancers	-	-	-	+	+	+	+	+	-	+	_	-	-
Urological cancers	-	-	-	+	+	+	+	+	-	+	_	-	-
Head and neck cancers	-	-	-	+	+	+	+	+	-	+	_	-	-

AL: Algeria, EG: Egypt, IQ: Iraq, JO: Jordan, KW: Kuwait, LB: Lebanon, MR: Morocco, OM: Oman, K.S.A: Saudi Arabia, SY: Syria, TN: Tunisia, UAE: United Arab Emirates, YE: Yemen,

N/A: non applicable

+: Item discussed in the country guidelines, -: Item non discussed in the guidelines
